# BRACAVENIR - impact of a psychoeducational intervention on expectations and coping in young women (aged 18–30 years) exposed to a high familial breast/ovarian cancer risk: study protocol for a randomized controlled trial

**DOI:** 10.1186/s13063-016-1642-4

**Published:** 2016-10-21

**Authors:** Fabrice Kwiatkowski, Pascal Dessenne, Claire Laquet, Jean-Pierre Daures, Mathilde Gay-Bellile, Yves-Jean Bignon

**Affiliations:** 1 Laboratoire d’oncologie moléculaire, Centre Jean Perrin, 58 rue Montalembert, 63011 Clermont-Ferrand Cedex, France; 2 UPRES 2425, Décision médicale personnalisée, Montpellier, France; 3 Mutualité Clinique Beau Soleil, Montpellier, France

## Abstract

**Background:**

Young women exposed to a high hereditary breast and ovarian cancer (HBOC) risk are particularly vulnerable. They are ignored by health prevention measures but exposed to a stream of contradictory information (medicine, media, Internet). They may feel concerned about surgical prevention issues at a key moment of their identity construction (self, relationship, sexuality). We designed a special psychoeducational intervention to help these women cope better with these difficulties.

**Methods/design:**

The BRACAVENIR study consists of a prospective, randomized superiority phase II trial with a wait list control group. Participants are childless young female counselees (aged 18–30 years) seen at the oncogenetics department of the Centre Jean Perrin and belonging to HBOC families either with or without *BRCA* mutations. They will be invited to attend a weekend group session at a spa resort and to participate in short expert conferences and focus group activities (group sharing, Moreno role game) supervised by a psychotherapist. Two sessions separated by a 6-month delay (wait list) will enable us to evaluate the intervention’s effect by comparing questionnaire scores between the 6-month time points. The main endpoint is an increase of the Herth Hope Index by at least 1 SD. Secondary endpoints are self-esteem, anxiety trait, anxiety state, coping, and quality of life. With a one-sided α = 0.05 and β = 0.20, 12 participants will be needed by group, plus an additional 2 in anticipation of dropouts. Participants will be randomized 1:1 to the first or the second session so that the groups will be comparable.

**Discussion:**

The intent of this trial is to bridge the gap on a psychosocial level in these young women with HBOC. A particularity of the design is the use of a waiting list, which should allow for avoiding major bias. The intervention consists of a short session that could be proposed to other young counselees if successful. The results may bring complementary information to facilitate the intervention and also influence the contents of the oncogenetic consultation.

**Trial registration:**

Ethics committee CPP SUD-EST-6: IRB00008526. Registered on 18 March 2016.

ClinicalTrials.gov identifier: NCT02705924. Registered on 2 March 2016.

**Electronic supplementary material:**

The online version of this article (doi:10.1186/s13063-016-1642-4) contains supplementary material, which is available to authorized users.

## Background

Carriers of mutations favoring the development of cancer in adulthood encounter various life difficulties. In addition to an increasing cancer risk with age, many questions arise concerning the future and how to build long-term projects. Although prevention and medical screening receive great support from health institutions, most psychosocial consequences remain unanswered. Among members of cancer-prone families, some are ignored by health systems, in particular the youngest women exposed to a high risk for breast and ovarian cancer. Indeed, these women are too young to be concerned with prevention measures. However, some of them have already been confronted by a mother’s disease and experienced their mothers’ early death. This early period of their lives also makes them vulnerable as they face identity issues, the onset of romantic relationships, or questions about how to plan for procreation [1, 2].

In parallel, these young women stand in the cross fire of an abundance of contradictory information spread by the media, websites, or internet forums. The rapid evolution of medicine adds to this cacophony in terms of possible prophylactic surgery, assisted procreation, embryo selection, and gene therapies, with discordant voices between specialists, even in Western countries. Unfortunately, prophylactic interventions directly address the intimate sphere of these young women who are still maturing.

In many families, the mutations responsible for cancer risk are known (mainly *BRCA1/2*), giving to descendants one of two chances of having a mutation. In other families, no specific mutation can be diagnosed, and it is impossible to know for sure whether a family member carries a mutation. Such uncertainty makes communication with relatives or friends more complex, in particular when one tries to transmit oncogenetic information [3]. Consequently, a possible reaction to this incertitude may consist in denial, with a probable reduction of prevention efficacy.

Socioeconomic difficulties are a known cause of refusal of medical care [4]. This is also true for cancer prevention. Such difficulties represent an additional burden for some, although most medical care in France is cost-free for patients.

It is therefore very important to propose new strategies to communicate with young counselees in oncogenetics. We thus developed a psychoeducational intervention tailored for these young women and decided to test its efficacy in a prospective phase II trial. In this article, we describe the content of the intervention and the method to provide evidence of its interest.

## Methods

### Objectives

The main objective of this trial is to change the expectations and improve the coping of young women exposed to a high risk of hereditary breast and ovarian cancer (HBOC). Changes of expectations have been chosen as the first objective, while coping modifications have been placed within the secondary objectives. We expect that, given the type of information that will be provided during sessions, expectations may vary faster than coping manners, the former seeming more sensitive to information renewal.

Other secondary objectives are:

- To promote a better understanding of oncogenetic information
- To study if socioeducational differences may limit overall compliance with medical screening and if better social communication may help prevent refusal of care
- To help these young women to build a more satisfying self-image
- To facilitate the way these young women resolve life issues such as marital relationships and procreation planning, and so to favor their emotional and sexual fulfillment
- To improve the chances of achieving a better long-term quality of life (QoL)
- To improve adherence to medical screening

### Psychoeducational intervention

The intervention will consist of a group session of a dozen participants at a spa center during a weekend. Participants will arrive on a Friday evening. After dinner, a television documentary about the story of a family with *BRCA* mutation will be shown. During the following 2 days, besides free spa activities available in the center, participants will attend ten small conferences lasting 15–30 minutes each. Experts will present the state of the art in various domains, including the following:

- Latest knowledge in oncogenetics (presented by an oncogeneticist possessing doctoral and medical degrees who is also an academic researcher in this domain)
- Recommendations and morbidity of prophylactic breast surgery and adnexectomy (presented by a surgeon specialist in gyneco-oncology)
- Psychological aspects in oncogenetics (presented by a clinician psychologist possessing a master of science degree)
- Epidemiology of HBOC and comparative mortality risks with other syndromes or life habits: how one can reduce cancer risk (e.g., tobacco, alcohol, breastfeeding) (presented by a statistician possessing a master of science degree)
- Good methods of breast cancer prevention (presented by a medical doctor who is a clinician senologist responsible for breast cancer screening)
- Assisted medical procreation and embryo selection (presented by a clinician possessing medical and doctoral degrees and specializing in this domain)
- Importance of nutrition and physical activity as risk modulators (presented by an academic researcher possessing medical and doctoral degrees and a nutritionist)
- Description of the assistance program developed to help counselees exposed to a high cancer risk (HBOC, Lynch syndrome) following their medical screening (presented by an oncogenetic counselor as well as a clinical nurse specialist)
- Health insurance facilities and social financial support (presented by a professional social worker)

For 15 minutes after each conference, participants will be able to comment on what they heard and/or to ask questions of the experts.

The remaining time after this preliminary information (i.e., about a half of the weekend) will be devoted to group activities, in particular role-playing games (Moreno psychodrama approach [5]) and group sharing under the supervision of a psychotherapist. The psychodrama will start with a short presentation of the rules: general empathy, no acting, and no after-session talking or gossiping about what happened or what was said, in particular naming participants, as well as silence when a role-play is ongoing. The second step asks participants to volunteer and stage a particular concern or difficulty that they would like to “treat.” If several persons volunteer, an election is undertaken to select the topic appearing most important to the majority of participants. If no one volunteers, the process starts with a “sticker game” with a theme such as “what I think of your mutation.” Each participant has to write on a small paper a short sentence (corresponding to the theme) describing what she may feel about another participant and sticks it on the latter’s forehead. Once all labels are stuck, all participants sit back in a circle and, one after another, each unglues her sentence, reads it aloud, and comments on it. Depending on the emotions raised during this exercise, a theme will be chosen for the psychodrama and role-played by participants under the regulation or direction of the psychotherapist. The selected participant explains the situation she selected, including the protagonists, the details of the event, and the difficulties she faces, and then chooses another participant to play her role. The stage director organizes the replay, promotes dialogue, invites standing in, enriches the drama, sheds light on dark zones, and proposes alternatives. If the game happens to be short, another “drama” can be introduced and played. About 3 h will be devoted to this exercise and the group sharing just afterward, leaving some time for spa activities before dinner.

We expect that the greatest effect of our intervention on expectations and coping will rely on how participants integrate the delivered information and how the exercises enable them to objectivize difficulties and suggest possible solutions. Such an intervention, if it is to be generalized, would induce numerous costs: the weekend session in a spa center (2½ days), experts’ fees, transportation of participants and experts, psychologists’ time, and organization costs. Overall, these costs would represent about $600 per participant (excluding data management, study design, and analysis).

### Study design

The study is a randomized, prospective, monocenter, psychoeducational phase II trial comparing the evolution of indicators between preintervention and 6 months postintervention. Two groups will be compared: a first group of asymptomatic participants who will attend the session immediately and a second group corresponding to the “waiting list” (control group). The waiting list session will occur 6 months later than that for the first group. Measured indicators will be obtained 2 weeks before and 6 months after the first intervention for all participants, whatever their allocation group (Table 1). This will be possible because the questionnaires will be completed by participants at home on a dedicated website. Groups will therefore be comparable, and the only difference between them will be limited to the intervention.

**Table 1:** Schedule of enrollment, interventions, and assessments

		Study period				
	Enrollment and allocation	Baseline values	Session 1	Main outcome values	Session 2	Secondary outcome values
Time points	April-May 2016	First week of June 2016	Third weekend of June 2016	First week of November 2016	Third weekend of November 2016	End of May 2017
Enrollment						
Answer to the invitation letter	X					
Consultation and informed consent	X					
Allocation to session 1 or 2	X					
Website learning	X					
Interventions						
Group session 1			X			
Group session 2					X	
Assessments						
Socioeconomic data	X					
Psychosocial questionnaires		X	X	X	X	X

In March 2016, the study was approved by the local ethics committee (CPP SUD-EST-6: IRB00008526) and registered with ClinicalTrials.gov (NCT02705924).

### Population

Participants must belong to HBOC families, be aged between 18 and 30 years, be single, and be childless without any personal history of cancer. They must have consulted at the oncogenetics department of the Jean Perrin comprehensive cancer center (CCC) and must been tested for the presence of germline *BRCA* mutations. If a familial germline *BRCA* mutation has already been diagnosed, these women must carry the familial mutation. If no known deleterious mutation has yet been found within the family, these women may be included if their familial risk is high (Eisinger score ≥6 or Manchester score ≥16) [6–8]. To limit travel costs, participants must live in the Auvergne region (central France). Participants must also sign an informed consent form before inclusion.

Exclusion criteria include pregnant women and those who cannot answer questionnaires either because of language difficulties or because they cannot write in French correctly. Counselees who cannot connect to the dedicated website will also be excluded, but this is not likely to happen. Psychiatric disorders and/or ongoing related treatments will also result in exclusion, as will any treatment incompatible with a 2-day stay in a spa center.

### Recruitment and planning

Since 1988, the oncogenetics department of Jean Perrin CCC has been collecting pedigree information of cancer-prone families. Today, more than 7000 families are registered in the database, which comprises more than 170,000 members overall. The birthdate and address of counselees are recorded, in addition to the type of cancer risk and various medical annotations (in particular cancer history of the family members).

Individuals meeting the inclusion criteria will be extracted from the database, and invitations will be sent by mail to a randomly selected subgroup of 60 counselees. The first 28 young women answering positively to this invitation will be asked to consult at the oncogenetics department to receive a detailed description of the protocol and sign an informed consent form, by which they will agree to participate in the trial. After their consent is obtained, the participants will be randomized for allocation to the first or second weekend session. They will also receive a training course to enable them to complete the questionnaires using the dedicated website. The overall organization of the trial is displayed in Table 1.

### Randomization

Participants will be randomized 1:1 to the first or second session (intersession delay = 6 months) using a minimization technique and stratified according to the existence or absence of a familial *BRCA* mutation. We assumed that this characteristic might induce variations in coping and expectations and thus alter the impact of the psychoeducational intervention if the studied population were not balanced with regard to this characteristic.

### Evaluation criteria

Endpoints and their evolution will be evaluated using self-questionnaires. To obtain relevant data, these questionnaires needed to match several criteria:

- Expectations, personality traits and coping strategies likely correlate, so a hierarchy between these psychosocial variables was necessary to prioritize our objectives. Many questionnaires could have been selected for investigation of the relevant psychological dimensions, but their number had to be limited to the necessary minimum so that the time for questionnaire completion would not take too much time and the rate of missing data would be acceptable.
- Because they are too young, participants that we accrue do not naturally focus on cancer and other health issues. We thus wanted to avoid as much as possible questionnaires that target mainly health domains.
- Questionnaires had to be validated and translated into French.

**Fig. 1:**
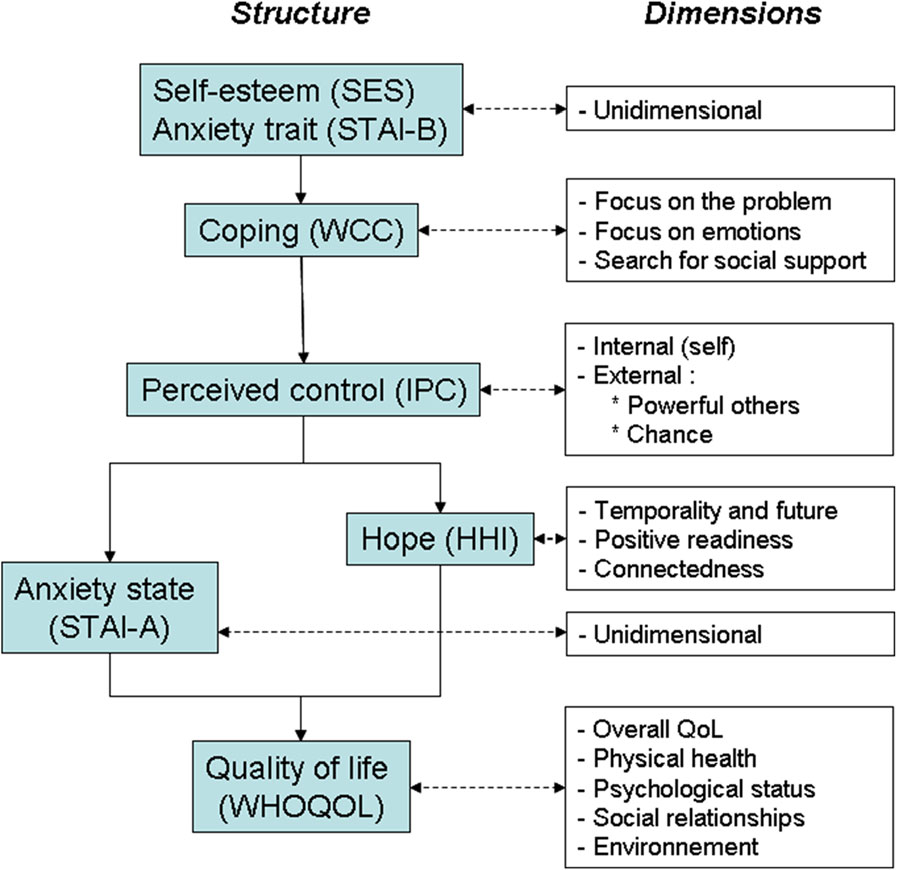
Construct of the psychological model enabling the evaluation of the study endpoints. *HHI* Herth Hope Index, *IPC* Internal, Powerful Others, and Chance Locus of Control Scales, *QoL* Quality of life, *SES* Self-Esteem Scale, *STAI* State-Trait Anxiety Inventory, *WCC* Ways of Coping Checklist, *WHOQOL* World Health Organization Quality of Life questionnaire

Figure 1 depicts the construct of our psychological model and the questionnaires used at each level.

The various endpoints evaluated in our protocol start from personality traits characterizing our young counselees that can be considered stable: self-esteem and trait anxiety belong to this category. We rejected any characterization of other general personality traits and/or psychiatric disorders, considering that such topics, too distant from our objectives, should be of limited influence on our endpoints (because of the study selection criteria) and would provide variables with too large a range of categories that our study would not efficiently handle (too small a sample size).

Two questionnaires evaluate personality traits: the Self-Esteem Scale (SES) [9] and the State-Trait Anxiety Inventory (STAI)-B for the rather stable anxiety trait [10, 11]. At the second level, coping is examined using the Ways of Coping Checklist (WCC), the ways of coping described by Folkman and Lazarus [12, 13]. Resulting from interactions and feedback between the individual and the environment, perceived control is a pivotal concept. Levenson [14] has shown that it has three aspects: a locus of control originating in the self, and another one located outside the individual with two possible sources: fate/luck and “powerful others.” For our counselees, we might wonder if HBOC predisposition is considered an inner or outer locus and if an unknown mutation could boost the “fate/chance” dimension. In young women, the locus of control may influence two further labile dimensions: anxiety (state) on the one hand, and expectations on the other. We will evaluate state anxiety using the STAI-A, while expectations will be explored with the Herth Hope Index (HHI) [15, 16]. The overall outcome of this construct in interaction with standard education and/or socioeconomic levels will be estimated using the World Health Organization Quality of Life (WHOQOL) generalist questionnaire [17]. All of these questionnaires are detailed below.

#### Herth Hope Index

With the HHI [15, 16], expectations can be split into two “spheres” [18]: first a factual, concrete level; second, a subjective one that includes optimism/pessimism, confidence, and the feeling that life makes sense. The first sphere includes the possible evolution of science that will perhaps someday discover remedies against cancers and genetic predispositions. We did not plan to investigate this sphere on which individuals have no influence. The second sphere is more interesting for our purposes: We expect that with adequate and positive information, the confidence in the future of our young women will grow and that they will be able to build projects more easily. Rustøen et al. [19] reported that, among newly diagnosed cancer patients, hope could be improved by using a short psychoeducational intervention, although the improvement for their patients did not persist 6 months later, nor did it seem to influence QoL. Our hypothesis is that such persistence in asymptomatic young adults may last 6 months or more, and this is our main endpoint.

The HHI fits this second sphere of expectations. It has demonstrated very good psychometric qualities, in particular among cancer patients, among whom hope appears to be a basic mechanism involved in coping [16]. Phillips-Salimi et al. [20] proved the validity of the HHI in adolescents and young adults with cancer. We think it is well adapted to our young participants in particular because it is not health-oriented; it will not echo in young women with HBOC a future possible disease and therefore will not risk stressing them uselessly. In a different domain, hope management has demonstrated some efficacy in the prognosis of cardiovascular disease [21]. This conclusion let the authors propose a five-step management plan to maintain hope within a transition framework: (1) acknowledging the changing of life circumstances, (2) restructuring reality, (3) dealing with vulnerability, (4) achieving normalization, and (5) resolving uncertainty” [22]. Our intervention will deal with the four last steps.

Another advantage of the HHI is its short format: It contains only 12 simple proposals quoted using a 4-point Likert scale, ranking from 1 = strongly disagree to 4 = strongly agree. Its internal structure is composed of three dimensions: temporality and future, positive readiness and expectancy, and social/spiritual connectedness [23]. Although we expect that our intervention may improve preferentially the first two dimensions, the global score will constitute our primary endpoint (sum of all 12 items, but items 3 and 6 reversed). This questionnaire has been translated and validated in French by Lafrance [24]. It will be used to evaluate the primary endpoint; the other questionnaires described below will be used to evaluate secondary endpoints.

#### Self-Esteem Scale

The SES [9] is a short, ten-item questionnaire that evaluates global self-worth by questioning positive and negative feelings about the self. The origin of self-esteem seems to be composite and may be searched in early childhood, parental models, and context favoring personal development (e.g., parental concern, education, sports participation). The unidimensionality of the scale seems demonstrated, although all items are not weighted equally in the construct analysis [25]. Self-esteem appears an important issue in our study because its level conditions whether individuals will seek social support when they are confronted with unsolvable problems [26]. This attitude may impact the way they take into account the messages delivered during our intervention and how they will adapt their life strategies thereafter. Self-esteem has also proven to influence the level of hope, with persons with the higher self-esteem entertaining more hope [27].

Answers are quoted using 4-point Likert scales ranging from “strongly agree” to “strongly disagree.” We expect that a familial cancer predisposition might lower self-appraisal and that a more positive regard of it might contribute to an increase in SES score. The SES has been validated in French with different samples, including people with schizophrenia [28].

#### State and Trait Anxiety Inventory (STAI-A and STAI-B)

The STAI [11] is a questionnaire widely used to evaluate anxiety in such a way it cannot be confused with depressive syndrome. It comprises 40 items rated on a 4-point scale from “almost never” to “almost always.” The first 20 items evaluate anxiety as a state, corresponding to a labile/contextual aspect. The last 20 items estimate anxiety as a personality trait, independent of the environment. In our study, we expect, after our psychoeducational intervention, a larger change of state anxiety than of trait anxiety. Also, we assume that a reduction in anxiety will correlate with an increase in hope (HHI score). The STAI has been translated into French and validated by Schweitzer and Paulhan [29].

#### Ways of Coping Checklist

Coping is a wide concept. It can be defined as either a personality trait (“characteristic ways of responding to changes of any type in the environment” [30]) or a behavioral characteristic (“a conscious, intentional, goal-directed response, tailored to the specific demands of a stressor” [31]). The WCC [12, 13] has been translated into French in several versions: by Vitaliano (42 items) [32] and, based on this first translation, one by Schweizer and Paulhan (29 items) [29] and one by Bruchon-Schweitzer (27 items) [33]. We decided to use this last one [33], validated in 2010 in cancer patients by Cousson-Gélie et al. [34] despite a reduced set of 27 items. In the French 27-item version, the 2 main dimensions have been confirmed, characterizing either the focus on the problem (10 items) or the focus on emotions (9 items). A third dimension has been found, consisting in the search for social support (8 items) [33].

#### Perceived control (Internal, Powerful Others, and Chance Locus of Control *Scales*)

Perceived control synthesizes the equilibrium between actions launched by the individual and the feedback returned by the individual’s environment. Levenson’s Internal, Powerful Others, and Chance Locus of Control Scales (IPC) [14, 35, 36] is one of the most often used questionnaires to analyze how individuals try to control their destiny. Locus of control can be perceived either (1) within the self or (2) outside the self with two origins: fate/luck and “powerful others.” Depending on the locus, an individual’s cognition and behaviors vary. In young women exposed to a high familial cancer risk, the locus may appear to be outside the self (a *BRCA* mutation is like a twist of fate). Our intervention will try to teach how, with adapted behaviors (i.e., mainly changes in life habits), overall cancer risk can be halved, meaning that a part of the locus of control dwells, in fact, within the individual. A low level of perceived control has been proven to be related to psychological disorders such as depression [37] as well as a low HHI [22]. The 24-item IPC has been translated into French by Loas et al. [38].

#### Quality of life (WHOQOL questionnaire)

QoL is the subjective result of the many interactions between individuals (including health status) and their environment. Because it constitutes the focal point of all these interactions, we thought it might be of interest to check how our intervention could influence QoL over the long run. The WHOQOL questionnaire [17] has been preferred to other validated scales (e.g., European Organization for Research and Treatment of Cancer Quality of Life Questionnaire Core 30 for Cancer [EORTC QLQ-C30] [39], 36-item Short Form Health Survey [SF-36] [40]) because it does not focus on health-related QoL. The version used in our study corresponds to the WHOQOL-BREF (26 items). This version was derived from the 100-item WHOQOL and showed similar properties. The authors suggested that this short version could advantageously replace the long version in trials and surveys investigating QoL [17]. It contains four main dimensions: physical health, psychological health, social relationships, and environment. These dimensions have been confirmed in the French translation by Leplège et al. [41].

### Methodology

#### Sample size

Sample size has been determined by taking into account two factors: first, the need for small groups for the sessions to be efficient (i.e., letting all participants get involved in the exercises and have time to share their emotions or opinions), and second, the need for the trial to have sufficient power to evidence differences. Our target is to objectivize differences between groups of at least 1 SD of the differential variation in the HHI questionnaire score between inclusion and 6 months. This hypothesis is compatible with the results published by Herth et al. [16], who found that, after a psychosocial intervention in cancer patients, the improvement to the global HHI score equaled 9 points with an SD of 9.9. An expected 1-SD difference may be questioned, however: Herth’s population corresponded to cancer patients aged between 20 and 80 years. Our population is healthy, evidently much younger, and includes only females. One of our expectations is that the study outcome invalidates or confirms if this reference was relevant; if not, this will help further studies to build a better-fitting design. With this 1-SD hypothesis, a one-sided α-risk equal to 0.05, and 80 % power (β = 0.20), at least 12 participants per group will have to be accrued. Considering that some participants may refuse at the last moment to come to the session, two more participants will be accrued per arm. Overall, 28 participants need to be included.

#### Data management

Data will be collected using an electronic case report form (eCRF). Capture System software (Clinsight, Cenon, France) will be used to allow participants to complete their questionnaires by Internet from home. They will be enabled to input data without the possibility of consulting their previous answers (questionnaires at inclusion or at sessions). This blinding is important to prevent both a back-correction of previous answers and also to limit the risk of a direct copy for participants who would avoid recanting. Data will be monitored according to the specifications defined for this kind of study; in particular, special attention will be paid to limit the amount of missing data. Two kinds of control will be performed using the software. First, the eCRF will automatically signal if a question is not answered. However, the participant keeps the right to confirm the missing item. Second, if an entire questionnaire is not completed, because this might be an omission, a message will be sent by the data manager through the mailing device of the software to ask the participant to complete the form or to confirm that she did not forget to fill the questionnaire but intentionally left it blank.

#### Data analysis

A statistical analysis plan will be written prior to conducting any outcome analysis, excluding queries to manage data. The statistical analysis will start after the database is locked. This will be done in two steps when the 6-month and then the 12-month questionnaires are collected.

Data analysis will begin with a description of population characteristics (age, known *BRCA* mutation, socioeconomic and educational categories). The balance of these characteristics will be checked by allocation group. Missing data will be analyzed by group, by type of questionnaire, and by time (inclusion, sessions, 6 and 12 months). Further description will present the longitudinal analysis of questionnaire scores over the course of 1 year. Differences related to socioeducational and socioeconomic categorical data and scores at inclusion will be analyzed using one-way analysis of variance (ANOVA) or Kruskal-Wallis H test. Overall convergent/divergent analysis between scores, independently of groups and times, will be verified by correlation analysis (Pearson’s or Spearman’s correlation if distributions are abnormal). A correspondences analysis will present the global description of the results and the relationships between questionnaire dimensions.

The main endpoint will be tested between allocation groups using individual HHI score variations (change at 6 months from time of inclusion) by Student’s *t* test or Mann-Whitney *U* test if normality/homoscedasticity is not achieved. All scores by group will be analyzed longitudinally using two-way ANOVA (mixed model) with either raw scores or their variation from baseline.

The statistical analysis will be performed using SEM software [42] and R version 3.0.1 software. The SPIRIT checklist, describing correspondances between the protocol and standard issues addressed in clinical trial, is provided as Additional file 1.

## Discussion

Prospective, randomized psychoeducational studies are often difficult to conduct. First, no blinding can be organized; interventions and group allocations are necessarily described in the informed consent form. Second, a particular difficulty is encountered when one tries to precisely define psychological endpoints; they are usually multiple, and they influence each other. Third, some endpoints vary very rapidly shortly after the intervention, but in a labile way, and a quick positive improvement may hide long-term inefficacy. However, developing an intervention without evaluating its effects would lead to a dead end.

To avoid selection bias, randomization using a waiting list is often the best solution. It ensures that participants are alike, and it places them on an equal footing. Unfortunately, if the waiting delay is too long, some of the participants allocated to the waiting list may withdraw for various personal reasons. We do not expect this to happen with our young women; in particular, at such an age, severe diseases are not that frequent, even in an HBOC context. Also, a cost-free stay in a spa center seemed to us attractive enough to limit this possibility. However, we have decided to increase the sample size a little so that such events would not reduce the study’s already limited power (80 %).

The hierarchical structure of our questionnaires is a second difficulty. The tendency to multiply the questionnaires used needs to be fought so that questionnaire completion is acceptable and can be performed in a short time. Especially if the completion has to be repeated over time, the missing data rate can increase, with consequences on estimates. Also, interactions between the various psychosocial dimensions explored cannot be circumvented easily and may induce confusion bias. Special attention must therefore be paid to both the complementarity of questionnaires and the hierarchical validity of studied dimensions. A careful examination of bibliography was performed, and several expert discussions between professional psychologists and methodologists were needed before we reached a consensus.

The lability of some psychosocial dimensions makes them useless to test the effect of an intervention. This was the case for Rustøen et al. [19] in their study of the impact of a psychoeducational intervention based on hope improvement in newly diagnosed cancer patients. It enabled them to significantly improve the Nowotny Hope Scale score after 2 weeks, but no gain was noticed after 6 months. Obviously, in these patients, if chemotherapy/radiotherapy is needed and/or if the surgery is ablative or handicapping, the consequences of the treatments nullify any benefit that might be gained using a short-term psychosocial intervention. Even without deleterious circumstances, short-term improvements that do not last are very frequent in our context. For example, a very strong postintervention decrease in Hospital Anxiety and Depression Scale [43] anxiety score was observed in another randomized trial testing a 2-week psychoeducational group intervention. That study addressed patients with breast cancer previously treated by adjuvant chemotherapy, and physical activity and nutrition were the main topics of the intervention [44]. This immediate improvement of anxiety measured after the intervention was followed by a return to basal values at 6 months, persisting for the long run. In the present study, our 6-month primary objective takes into account an assumed stability of expectations over such a delay in the absence of intervention and a persistence of the improvement due to the session—if any—lasting at least 6 months. In our context, if improvement of scores would last for a shorter time, the intervention would be of low interest. The 1-year evaluation constitutes a secondary endpoint that will enable us to verify whether the 6-month results are maintained.

Another discussed subject was the “good” time to start evaluating the intervention. The baseline measure was initially placed at inclusion. Because the accrual period will last about 2 months, however, the 6-month delay would not be respected for participants included early. We thus decided to shift the questionnaire self-completion at a fixed time point 2 weeks before the first session for everybody. Meanwhile, as the provision of socioeconomic data at inclusion might be a good opportunity to teach the participants how to use our dedicated website, we decided to reserve a moment after obtaining the consent signature to explain how to enter the site and complete the forms. The evolution of the other estimates and the cross-correlation analysis of questionnaire dimensions will help us to determine if another aspect should be used instead of the focus on “hope” to improve, with better efficacy, the overall QoL of our young women.

Finally, we as yet have no idea how our proposal will be perceived by our young asymptomatic women. The rate of refusal and the reasons by which they will justify their not entering the study will be analyzed and will help us develop better prevention strategies for the youngest counselees. One of the purposes of this phase II trial is to verify the feasibility and acceptability of the intervention to participants, evaluate its effect size on the various scales, and prepare a larger national phase III trial based on a more solid hypothesis. The acceptability is of major importance, and a better definition of the young population that we target may need some adjustments. In particular, the birth of one child does not eliminate worries regarding new procreation projects; perhaps it even increases them as questions about the long-term security of the child (Will I live long enough to ensure his future?) are suddenly raised by his arrival. So, the inclusion criteria of our pilot study will probably need to be widened in a further trial to include young women facing more concretely not only the transmission dilemma of their mutation but also familial existential issues.

### Trial status

The trial was not started when this article was written (October 18^th^, 2016).

## Additional file

Additional file 1: Standard Protocol Items: Recommendations for Interventional Trials (SPIRIT) 2013 checklist. (DOC 120 kb)

## Abbreviations

ANOVA: Analysis of variance
CCC: Comprehensive cancer center
eCRF: Electronic case report form
HBOC: Hereditary breast and ovarian cancer
HHI: Herth Hope Index
IPC: Internal, Powerful Others, and Chance Locus of Control Scales
QoL: Quality of life
SES: Self-Esteem Scale
STAI: State-Trait Anxiety Inventory
WCC: Ways of Coping Checklist
WHOQOL: World Health Organization Quality of Life questionnaire
WHOQOL-BREF: 26-item World Health Organization Quality of Life questionnaire
EORTC QLQ-C30: European Organization for Research and Treatment of Cancer Quality of Life Questionnaire Core 30 for Cancer
SF-36: 36-item Short Form Health Survey
